# Social Vulnerability Indicators for Flooding in Aotearoa New Zealand

**DOI:** 10.3390/ijerph18083952

**Published:** 2021-04-09

**Authors:** Kylie Mason, Kirstin Lindberg, Carolin Haenfling, Allan Schori, Helene Marsters, Deborah Read, Barry Borman

**Affiliations:** Environmental Health Intelligence New Zealand, College of Health, Massey University, P.O. Box 756, Wellington 6140, New Zealand; k.lindberg@massey.ac.nz (K.L.); c.haenfling@massey.ac.nz (C.H.); A.schori@massey.ac.nz (A.S.); t.h.marsters@massey.ac.nz (H.M.); d.read@massey.ac.nz (D.R.); b.borman@massey.ac.nz (B.B.)

**Keywords:** social vulnerability, resilience, indicators, natural hazards, flooding, disaster, health

## Abstract

Social vulnerability indicators are a valuable tool for understanding which population groups are more vulnerable to experiencing negative impacts from disasters, and where these groups live, to inform disaster risk management activities. While many approaches have been used to measure social vulnerability to natural hazards, there is no single method or universally agreed approach. This paper proposes a novel approach to developing social vulnerability indicators, using the example of flooding in Aotearoa New Zealand. A conceptual framework was developed to guide selection of the social vulnerability indicators, based on previous frameworks (including the MOVE framework), consideration of climate change, and a holistic view of health and wellbeing. Using this framework, ten dimensions relating to social vulnerability were identified: exposure; children; older adults; health and disability status; money to cope with crises/losses; social connectedness; knowledge, skills and awareness of natural hazards; safe, secure and healthy housing; food and water to cope with shortage; and decision making and participation. For each dimension, key indicators were identified and implemented, mostly using national Census population data. After development, the indicators were assessed by end users using a case study of Porirua City, New Zealand, then implemented for the whole of New Zealand. These indicators will provide useful data about social vulnerability to floods in New Zealand, and these methods could potentially be adapted for other jurisdictions and other natural hazards, including those relating to climate change.

## 1. Introduction

Natural hazards can have major impacts on people’s health and wellbeing, both in the short and long term, and through both direct and indirect impacts from the hazard. Managing disaster risk, and protecting the health and wellbeing of populations from natural hazards, is becoming increasingly important in this changing world. Climate change is likely to increase the frequency and severity of climate-related disasters such as floods in many regions of the world [1]. Additionally, population growth, urbanisation, and changing socioeconomic conditions can increase society’s exposure and vulnerability to natural hazards [2]. In this context, having a clear understanding of the likely impacts of disasters on people’s health and wellbeing, and who is the most vulnerable to these impacts, can help support the planning and targeting of interventions [3].

In Aotearoa New Zealand, the most frequent and costly natural disaster is flooding. Approximately two-thirds of the New Zealand population live in flood-prone areas [4], and many of New Zealand’s towns and cities are built on floodplains. On average, a major flood occurs every eight months in New Zealand [4], and the total costs of flooding in New Zealand are estimated to be more than $125 million per year [5]. In the ten year period 2009–2018, there were 28 flood-related events where insurance damages were more than $1 million (inflation adjusted) [6]. In New Zealand, floods have had substantial impacts on people’s lives, in both the short term (e.g., gastrointestinal illness), as well as longer term (e.g., psychological distress, displacement) [7,8,9,10]. Flooding is likely to increase in frequency and severity in New Zealand due to climate change [4], and is one of the key risks from climate change this century in New Zealand [11].

Flooding can have a range of health impacts, including drowning, trauma injuries, hypothermia, and infections, as well as electrical injuries, burns and explosives injuries [12,13,14,15,16]. Floods can also increase the risk of waterborne diseases such as gastrointestinal illnesses, hepatitis A and E, cholera, typhoid fever, leptospirosis, and vector-borne diseases [12,13,15] and can increase the risk of heart attacks and respiratory problems [14], poor pregnancy outcomes [12,17], poor mental health [17], and exacerbations of substance abuse issues [17]. Longer term, flooding can impact on food supply, and lead to food insecurity and poor nutrition [12].

Infrastructure breakdown and disruptions to essential infrastructure and services during and after floods can also have substantial health impacts [17]. Disruptions to transport systems can delay first responders, impact on evacuation, and prevent access to health services, which can affect people with physical and mental health issues [17], including those on opioid substitution treatment [18]. Power outages can lead to food-borne illnesses through lack of refrigeration, carbon monoxide poisoning from unventilated generators, and affect medically dependent people, the young, and the old [15,17]. Floodwaters can also cause property damage, which can lead to overcrowding and displacement, and respiratory issues from damp and mouldy housing [19,20]. Flooding can also lead to loss of employment, lack of access to childcare services and schooling, and increased domestic violence [21,22].

The negative impacts of natural hazards such as floods on people’s health and wellbeing are not evenly distributed throughout society. People who are already struggling financially, who live in poor-quality housing, or who are socially isolated are more likely to experience significant adverse impacts from natural hazards [16,23,24]. Furthermore, not everyone in the population is able bodied, can hear, see and move, can understand the hazard, and can understand and carry out what they need to do to prepare or escape the hazard [25]. As a result, some population groups are more vulnerable to natural hazards and are less able to prepare for, cope with, and recover from natural hazards than others. Nonetheless, if community and government services are equitable and accessible before, during and after a disaster, more vulnerable people can have the same opportunity as others to be resilient [25]. Similarly, fully considering the needs of specific population groups in emergency management planning can reduce their vulnerability to natural hazards [23,26].

The term ‘social vulnerability’ is broadly used to refer to pre-existing conditions, characteristics or circumstances of people that affect their ability to prepare for, respond to, and recover from natural hazards [27]. A similar concept is ‘resilience’, which refers to the ability to “anticipate and resist the effects of a disruptive event, minimise adverse impacts, respond effectively post-event, maintain or recover functionality, and adapt in a way that allows for learning and thriving” [28], which can occur at the level of individuals, communities, organizations and/or states [29]. Information on social vulnerability is useful for risk assessment, understanding where the greatest need may be before, during and after a natural hazard, and providing insight about the needs of the local people and the likely impacts that natural hazards may have [25]. Social vulnerability indicators can therefore inform disaster risk reduction, preparedness, mitigation plans, and response and recovery activities, for civil defence practitioners, health authorities, local authorities, emergency services, and community organizations.

Several sets of social vulnerability indicators for natural hazards exist, including the Social Vulnerability Index (SoVI) [27,30], the Social Determinants of Vulnerability Framework [25], and the Social Vulnerability Index for Disaster Management [23] in the United States. Additionally, flood-specific social vulnerability indicator sets include the Urban Municipality Flood Vulnerability Index in Brazil [31], the Social Flood Vulnerability Index in England and Wales [32], and the Cologne (Germany) flood indicators [33]. Populations groups identified as being most vulnerable to floods, and/or natural hazards more broadly, include children, older adults, women, people with pre-existing health conditions, people living in poverty, low-income households, low educational attainment, unemployment, single parents, disabled people, certain races or ethnic groups, institutionalised populations, nursing home residents, renters, people with shorter length of residence, housing stock, poor quality housing, non-car ownership, household crowding, living in rural communities, and having English as a second language [7,23,25,27,31,32,33,34,35]. However, despite the numerous pieces of work on indicators of social vulnerability and resilience to floods and natural hazards more broadly, there is no definitive set of social vulnerability indicators or single approach to indicator development [34,36].

There are several challenges in developing social vulnerability indicators for natural hazards. Existing indicator lists and data sources may be difficult to transfer to a different setting (e.g., country to country), as indicator sets need to be context specific and relate to the social environment within that country [3]. Consequently, using ‘off the shelf’ indicator sets, or simply replicating indicator sets from other countries, may not work well in a country-specific setting. Furthermore, considering only a narrow set of impacts (e.g., only deaths and injuries) when scoping the social vulnerability indicators can miss important aspects such as housing, social connectedness, and economic wellbeing [35]. Additionally, some statistical methods (such as principal components analysis) used to develop indices and/or indicator sets can be influenced by data availability and the weightings used, leading to certain types of indicators (such as economic factors) having greater influence [34]. These statistical methods can also be challenging for policymakers to understand, and problematic to replicate, as the outputs are only pertinent to the time period and geographical area covered by the analysis. While summarising indicators into a single index value can provide simplicity of interpretation, it can also hide the underlying reasons for vulnerability in a local area [34].

In New Zealand, relatively few indicator sets relating to social vulnerability and natural hazards have been developed. Studies have tested the use of an index of neighbourhood socioeconomic deprivation (NZDep) as a proxy for vulnerability to natural hazards [37], proposed a set of earthquake social vulnerability indicators [35], and explored potential vulnerability indices for flooding in the Hutt Valley, New Zealand [34]. However, no set of social vulnerability indicators for natural hazards or flooding has been fully developed and implemented in the New Zealand context. While emergency management organizations well understand the factors that shape social vulnerability to natural hazards, there is little quantitative information at the local level to help inform their emergency management activities.

This study developed a set of social vulnerability indicators for flooding in Aotearoa New Zealand. These indicators aimed to provide a practical tool to inform local efforts in emergency management and health care, before, during and after a flood, and provide information to inform important risk reduction and emergency management activities.

The specific aims of this study were:

1. To identify a set of social vulnerability indicators for flooding for New Zealand
2. To identify populations vulnerable to flooding, and important facilities and infrastructure within flood zones, for a case study of Porirua City, New Zealand
3. To identify how indicators could potentially be used by emergency management, local councils and the health sector.

## 2. Existing Vulnerability Frameworks and Indicators

### 2.1. Conceptual Models for Vulnerability

In natural hazards research, risk is generally conceptualised as the potential for loss, and is given as a function of hazard and vulnerability, where vulnerability refers to the propensity of exposed people to experience harm and suffer loss [33]. Disaster risk has also been defined as a function of hazard, exposure, and vulnerability/capacity [38,39], while the Intergovernmental Panel on Climate Change (IPCC) has defined climate change risk as a function of hazard, exposure and vulnerability [40]. Thus, vulnerability is often a core component of disaster risk.

Some models already exist for understanding and describing different aspects of social vulnerability to natural hazards. The Hazards-of-Place model focuses on how the geographic context interacts with the social characteristics of society to produce the overall place vulnerability [30]. The Pressure and Release model describes how vulnerability arises from inequalities in society, which create pressure in society [41]. The Access model focuses on the access that people have to capacities, assets and opportunities [41]. The Vulnerability Framework frames vulnerability as having components of exposure, sensitivity and resilience, and being influenced by a range of contextual factors at the societal and environmental levels [42]. These models are not generally conflicting; rather, they describe vulnerability from different perspectives and have different focuses [33]. However, they generally do not explicitly consider the impacts of climate change.

For climate change vulnerability, the IPCC describes how vulnerability and risk are influenced by the hazard, exposure, sensitivity, and people’s capacity to cope and adapt [43]. Similarly, the US Climate Change and Health framework for determinants of vulnerability of human health to climate change includes exposure, susceptibility and adaptive capacity [17].

The Methods for the Improvement of Vulnerability Assessment in Europe (MOVE) framework has been developed as a generic conceptual framework for guiding vulnerability assessment and development of indicators [33]. The MOVE framework takes a holistic approach, being designed to work for not only natural hazards but also climate change. It incorporates the key elements of risk, hazard and vulnerability [33]. According to the MOVE framework, hazards (natural events or socio-natural events) interact with society (which includes vulnerability), to produce a risk (economic, social or environmental potential impact). In the MOVE framework, vulnerability has three components [33]:

- Exposure: temporal and spatial;
- Susceptibility/fragility: the predisposition to suffer harm; can include physical, ecological, social, economic, cultural, and institutional;
- Lack of resilience: lack of capacity to anticipate, cope and recover.

The MOVE framework has been tested in a number of case studies, including for flooding in Cologne (Germany) [33], Tokyo (Japan) [44] and Côte d’Ivoire [45].

The MOVE framework can be used to consider different types of vulnerability, including social, physical, ecological, economic, cultural and institutional. The social dimension of vulnerability in the MOVE framework refers to the propensity of people’s wellbeing to be damaged [33].

### 2.2. Existing Indicators for Social Vulnerability to Flooding

A range of social vulnerability indicators for natural hazards have been developed internationally. Some indicator sets or indices have been specifically developed for flooding, while others have been developed for natural hazards more generally (see Appendix A for more details).

One of the first social vulnerability indices for natural hazards was the Social Vulnerability Index (SoVI) [27,30]. The SoVI was based on the Hazards-of-Place model of vulnerability, and used statistical methods to reduce 42 variables to a key 11 variables. These variables were then weighted and combined to create the index, at the United States county level. The variables were a mix of demographic characteristics, built environment, and infrastructure characteristics.

In the United States, the Social Determinants of Vulnerability Framework identified key social factors that resulted in people having disproportionate exposure to risk and a decreased ability to avoid or absorb potential losses [25]. A grounded theory approach was carried out, using a link analysis of social factors from existing literature, to investigate the relationships between social factors. Seven key interrelated social factors were identified: children, older adults, people with disabilities, chronic and acute medical illness, social isolation, low-to-no income, and people of colour. Additional indicators that were also found to be important included women, lower educational attainment, limited English proficiency, renters, and a lack of a vehicle.

Several sets of social vulnerability indices for floods exist. The Urban Municipality Flood Vulnerability Index identified key components of urban vulnerability to floods in Brazil, incorporating age, health status, education, income, work status, access to telecommunications, housing, flood preparedness, and access to services [31]. The Social Flood Vulnerability Index, developed in the United Kingdom, included indicators about financial deprivation, pre-existing health problems, single parents, and the elderly [32]. The Social Vulnerability Index for Disaster Management created an index for the United States, based on 15 indicators across four social vulnerability domains: socioeconomic status, household composition and disability, minority status and language, and housing and transportation [23].

In the New Zealand context, one study tested the use of the New Zealand Index of Socioeconomic Deprivation (NZDep) [46] to understand vulnerability due to limited financial resources, as a proxy for social vulnerability to natural hazards [37]. However, the study noted that NZDep did not tell the full story of vulnerability, and could not act as a proxy for all types of vulnerability, such as age or disability status. A comparison of potential vulnerability assessment methods for flooding in the Hutt Valley (in the Wellington region of New Zealand) identified 38 initial proxy indicators relating to vulnerability to flooding, in the broad groupings of demographic, social and economic indicators [34]. This study tested different methods for combining indicators into indices, and concluded that different methods gave different picture of vulnerability. They therefore recommended that a comprehensive vulnerability assessment was required to fully understand vulnerability to floods, rather than simply an index. Subsequently, Kwok identified potential social vulnerability indicators for earthquakes in New Zealand, using the SoVI index as a starting point for indicators [35]. The suggested indicators covered poverty, race and ethnicity, wealth, age, gender, and care dependency, medical disability and health care access. The final indicator set was not implemented, as the study did not identify any data sources or indicator definitions, or make an indicator dataset available. The study concluded that social vulnerability indicators need to be context sensitive, and that future indicator development work could consider community objectives, such as knowledge and skills, economic wellbeing, housing, health, safety, social connectedness, civic participation, and population dynamics. Other studies in New Zealand have investigated factors relating to resilience to natural hazards, including describing resilience factors among New Zealand’s indigenous people, Māori, after the 2011 Christchurch earthquake [47], developing a national resilience index (incorporating system-level resilience factors) [48], and identifying resilience factors within selected communities, using qualitative research methods [49].

In summary, there is currently no consensus on the specific variables to use when measuring social vulnerability to natural hazards. However, some key socio-demographic groups appeared across multiple indicator sets, including young children, elderly, people with chronic health conditions, people with disabilities, low income, access to transport or communications, housing (rental housing, housing quality, overcrowding), occupation, and race or ethnicity. Many international indicator sets were also context specific, for example including indicators on the presence of informal settlements (e.g., slums and mobile homes), which may not be relevant to all countries. Additionally, a variety of methods have been used to develop indicator sets, with few studies using a conceptual framework to understand the underlying reasons why people might be vulnerable to flooding, or to guide indicator selection. Of those that did, the MOVE framework was the most common, identifying indicator for exposure, susceptibility and lack of resilience [33,45]. Other indicator sets used more generic dimensions, such as demographic, social and economic factors.

Given the scarcity of information and data about vulnerable populations in New Zealand, our study aimed to fill this gap by developing a set of social vulnerability indicators for flooding in New Zealand. Rather than attempting to replicate indicator sets developed in different jurisdictions and social contexts, which may not have been relevant for New Zealand, we chose to use a concept-driven approach for developing our indicator set. In addition, we chose to develop a set of indicators rather than an index, given the limitations of indices.

## 3. Materials and Methods

This study developed a set of social vulnerability indicators for flooding for New Zealand, then used a case study approach to test the indicators. An established indicator development process was used in the selection, design and implementation of the indicators.

### 3.1. Study Area

New Zealand is a geographically isolated island nation of approximately 5 million people, with many natural hazards, including floods, earthquakes, droughts and volcanoes.

A case study area of Porirua City was used to test the social vulnerability indicators. Porirua City is a territorial authority of approximately 180 square kilometres in size, located approximately 25 kilometres north of the capital city of Wellington, in the lower North Island of New Zealand. Porirua had an estimated population of 56,800 people in 2018.

**Figure 1:**
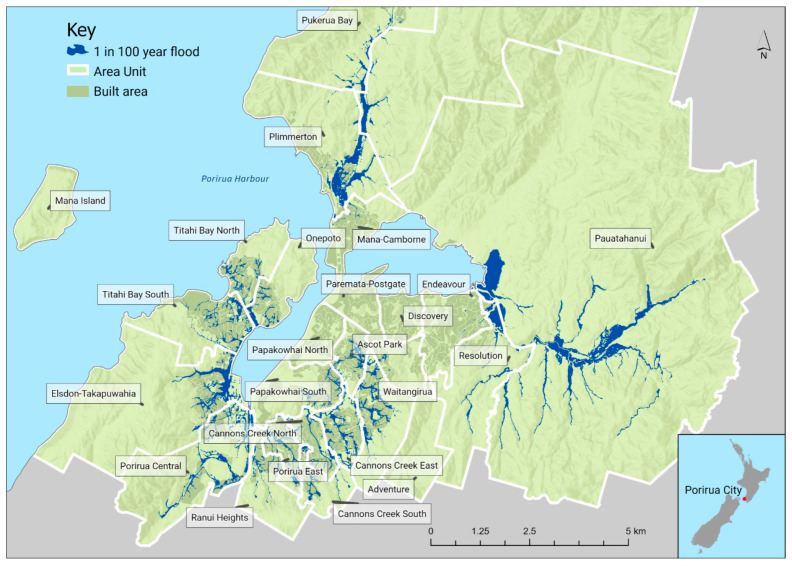
Map of Porirua City, with flood hazard zones and 2013 area unit boundaries. Note: Flood hazard zones give the flood hazard for a 1-in-100 year flood event. These hazard zones account for 100 year climate change impacts, in terms of sea level risk and increased rainfall. The models have used 1 metre sea-level rise and a 20% increased rainfall. The models were prepared by Wellington Water. Sources: ESRI, NASA, NGA, USGS, OpenStreetMap, Statistics New Zealand, Wellington Water, Porirua City Council.

Porirua has been affected multiple times by flooding, including in 2015 [50] and 2016 [51], which led to road closures, school closures, and flooded properties. In particular, flooding has affected the low-lying neighbourhood of Takapūwāhia, which has a marae for the local Māori iwi, Ngāti Toa Rangatira, that has come close to flooding on multiple occasions. Figure 1 shows the Porirua area with flood zones, with the area units of Cannons Creek, Waitangirua, Elsdon-Takapūwāhia, Porirua East, Ascot Park, Porirua Central, and Titahi Bay particularly affected.

### 3.2. Indicator Development Process

A robust and well-tested process for developing indicators was used. This indicator development process was adapted from methods for developing environmental health indicators [52], based on the World Health Organization’s process for developing children’s environmental health indicators [53] and Statistics New Zealand guidance for developing indicators [54]. Methods for developing environmental health indicators are relevant for this work, given their focus on the impacts of environmental hazards on human health and wellbeing, and the social, economic and demographic context in which these impacts occur.

**Figure 2:**
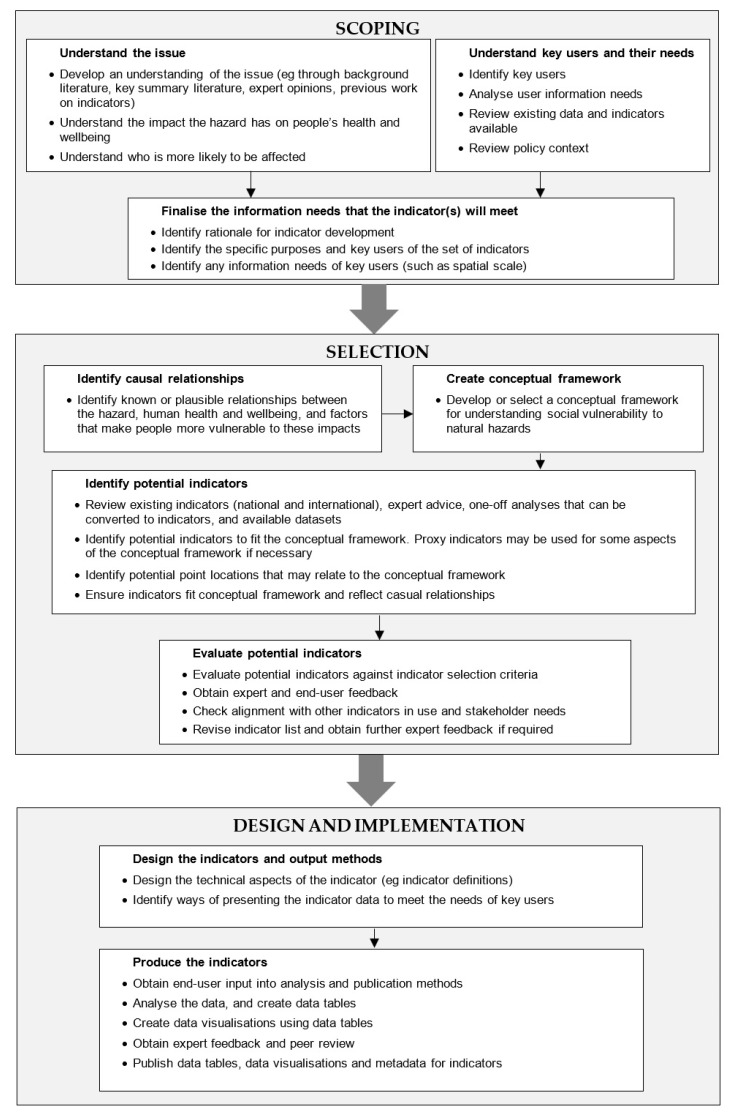
Social vulnerability indicator development process (adapted from Mason et al. [52]).

The indicator development process followed three major stages: scoping, selection, and design and implementation (Figure 2). Stakeholder engagement was included through the indicator development process to gain the perspective of experts and potential end users, with the Porirua geographical area used as the focus. The stakeholder group comprised representatives from the national emergency management agency, natural hazards researchers, as well as local Porirua representatives from the regional emergency management group, local council, public health service, and local health providers. Using this case study approach and engagement with stakeholders enabled identification of information needs of end users, feedback on the conceptual framework and proposed indicators, and identification of how the indicators would be used and best presented.

#### 3.2.1. Scoping Stage

The scoping stage defined the information and data needs for the indicators, through identifying the end users, indicator purposes, and key issues that the indicators needed to cover. For this study, impacts of flooding on human health and wellbeing, and factors that increase people’s vulnerability to these impacts, were identified through reviewing background literature and key summary literature, expert opinions, and previous indicator sets. Previous conceptual frameworks for social vulnerability, and social vulnerability indicator sets, were also reviewed. This stage also defined the study scope, and the world view and understanding of the issues that the indicators needed to reflect. A public health perspective was used to guide the study, and a strengths-based approach was incorporated where appropriate. The study was limited to individual-level factors influencing social vulnerability, rather than system-level indicators.

Potential end users of the indicators were also identified, including in the fields of emergency management, local and central government, and the health sector. Key needs for end users included having indicator data at the most detailed geographic level possible, to ensure that the vulnerability of local neighbourhoods could be understood. Data needed to be up-to-date, and relevant and meaningful to emergency management activities and the New Zealand context. Data were also needed about both the relative impact of vulnerability in an area, and the actual number of people affected. The policy context was also reviewed to identify how the indicators could be used within the existing emergency management structure in New Zealand.

#### 3.2.2. Selection Stage

The indicator selection stage identified a set of potential indicators that met the data and information needs found in the scoping stage. A fundamental approach for this stage was to use a conceptual framework to guide indicator selection [54]. Conceptual frameworks summarise concepts and how ideas are organized and relate to one another. Without a conceptual framework to guide indicator selection, indicator sets can be an eclectic mix of data-driven indicators, that make little sense together [54] and that are not balanced or relevant to the important underlying issues [53].

Our conceptual framework was developed for social vulnerability to natural hazards, based on existing frameworks of social vulnerability and resilience to natural hazards and climate change [17,33,55]. The framework was designed to be practical, with dimensions that were measurable and able to be used for guiding indicator selection, as well as taking into account climate change. Potential indicators were identified to measure the different dimensions of the conceptual framework, based on previous indicator sets, expert advice, and available datasets. A public health perspective was incorporated in the indicator selection process, with indicators focusing primarily on those indicators relevant to the potential impacts on people’s health and wellbeing. The indicators were then evaluated against ten indicator selection criteria, based on previous statistical guides [54] and the EHINZ programme [52], and adapted to meet the specific needs of the end users (Table 1).

The decision on the final set of indicators involved an iterative process of identifying potential indicators, assessing the indicators against selection criteria, and testing the indicators with stakeholders to ensure their usefulness.

#### 3.2.3. Design and Implementation Stage

The design and implementation stage included defining the indicators in detail, gathering and analysing the data, and preparing the final indicator outputs for end users. Considerations included the type of data output (for example, counts, rates, or summary indices) and geographical output (national, regional or local), depending on the needs of end users. In this stage, we developed a draft set of indicators, which were then visualised on maps for our case study area of Porirua, and assessed by stakeholders. Their comments and feedback were incorporated, and a final indicator set was created for the whole of New Zealand. Indicators were output at a range of geographical scales where possible, including meshblock (approximately 100 people), area unit (approximately 2000 people), territorial authority (of which there are 67 in New Zealand) and district health board (20 in New Zealand). Indicator data were output as both percentages of the population (to show relative impact) and counts of people (to show the number of people affected). In addition to the indicators, point locations relating to vulnerable populations (such as schools, rest homes and hospitals) were also identified through discussions with stakeholders, and guided by the conceptual framework.

The presentation of indicator outputs was also highly important, as indicator data needed to be accessible and meaningful to end users, and synthesise and communicate information, ideally in an informative and lively way [53]. Indicator datasets were produced in Microsoft Excel, and a range of dissemination methods for the indicator data were tested with stakeholders. As a result, ‘heatmaps’ were produced, by area unit for each local council, to show indicators for each social vulnerability dimension, coloured according to their relative value within the local council area. Online interactive maps and data visualisations were also created for Porirua with ESRI ArcGIS Story Maps and Tableau, showing maps of the indicators, as well as maps of point locations in relation to local flood hazard zones (incorporating 100 year climate change impacts). Metadata were also prepared for the final indicators, to provide detailed information about the indicators for end users.

#### 3.2.4. Stakeholder Engagement and Identifying Potential Uses for the Indicators

Stakeholders provided feedback and input at various stages throughout the indicator development process, including on the study scope, their data needs, the conceptual framework, draft indicators, and indicator data visualisations. Three stakeholder workshops were held throughout the study, including one where stakeholders brainstormed ways that indicators could be used in the local context. We also met with members of the local Māori iwi (tribe), Ngāti Toa Rangatira, to discuss flooding impacts on their community and vulnerabilities and barriers to feeling resilient to flooding, and received their input on the study through an iwi representative and facilitator.

## 4. Results

### 4.1. Developing a Conceptual Framework for Social Vulnerability

**Figure 3:**
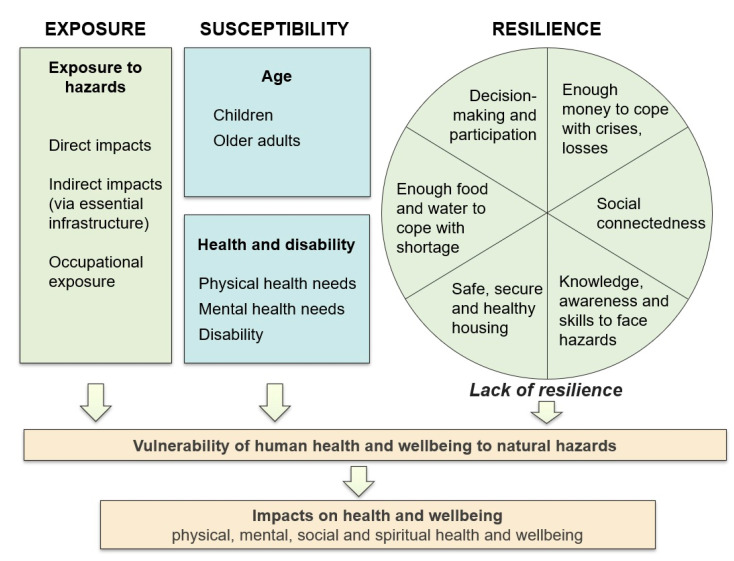
Conceptual framework for social vulnerability to natural hazards (own figure, based on conceptual models by Birkmann et al. [33], USGCRP [17], Wisner et al. [46,55] and Durie [57]).

We chose the MOVE framework as the basis for our conceptual framework to guide indicator selection, given its generic and holistic approach, its applicability to both natural hazards and climate change, and its proven utility as a framework to guide indicator selection. Therefore, our model incorporated the key elements of (i) exposure, (ii) susceptibility, and (iii) lack of resilience (Figure 3), from the MOVE framework. These key elements can be described as follows.

- Exposure: Exposure refers to exposure to the hazard in both time and space, and can include direct, indirect and occupational exposure.
- Susceptibility: We adopted the environmental health understanding, that people who are susceptible have a higher likelihood (or severity) of health impacts due to exposure to a hazard, compared with other people exposed to the same hazard [56]. Susceptibility can include innate susceptibility (largely due to genetic predisposition or physiology, such as children not having a fully developed immune system) and acquired susceptibility (through old age and/or illness) [56].
- Lack of resilience: In the context of social vulnerability, a ‘lack of resilience’ was interpreted at the individual level, to consider people’s individual capacity to anticipate, cope and recover. For this, we incorporated the ‘circle of capacities’ [55]. Capacities can be understood as the assets and resources that people have (and are able to use), to prepare for, cope with, and recover from disasters [55]. The circle of capacities model shows a circle with six components: enough money to cope with crises/losses (economic resources), solidarity (social resources), strength, knowledge and skills to face hazards (human resources), safe housing and infrastructure (physical resources), enough food and water to cope with shortage (natural resources), and decision-making power (political resources) [55]. A lack of these capacities or resources may increase a person’s vulnerability to natural hazards. These dimensions also align with practical functions and focuses of emergency management, such as providing financial assistance after a disaster. The circle of capacities was adapted for the New Zealand situation, with the input of stakeholders (Figure 3).

To reflect the risk to health and wellbeing risk from flooding, we used a holistic view of health, based on the Māori indigenous model of health, ‘Te whare tapa whā’ (the house of four walls), which holds that hauora (wellbeing) is made of the four aspects of physical, mental, spiritual and social wellbeing [57]. This is similar to whole-life views of health of indigenous peoples in other countries, such as Australia [58] and Canada [59], as well as the World Health Organization’s definition of health as ‘a complete state of physical, mental and social wellbeing, and not merely the absence of disease or infirmity’ [60].

Table 2 provides the rationale for each dimension of the framework, in terms of why people are more vulnerable to natural hazards.

Comparing our framework to other sets of social vulnerability indicators suggested that the dimensions aligned well with previous sets of social vulnerability indicators (see Appendix A). Common indicator topics across the sets of social vulnerability indicators included children, older adults, people with existing health conditions and/or disabilities, poverty, housing issues, and social isolation.

Certain indicators from previous sets did not easily fit into the identified social vulnerability dimensions of our framework. Indicators about ‘race’ and ‘ethnicity’ [25,27] are likely to represent marginalised groups who are more likely to lack political power and experience social and economic racism [23], which can increase their vulnerability via one or more of the dimensions in our framework. Similarly, while some indicator sets included ‘women’ as an indicator, this depends on the context; women can experience aspects of both vulnerability and resilience, therefore gender is not necessarily predictive of vulnerability [16]. Additionally, some indicator sets included community-level, structural or contextual factors in society, such as existence of early warning systems, single-sector economic dependence, infrastructure dependence, hazard planning, and land use planning, which did not refer directly to individuals or specific population groups.

### 4.2. Identifying Indicators and Data Sources

Potential social vulnerability indicators for flooding in New Zealand were then identified to represent all the dimensions of social vulnerability in the framework. A final set of indicators were identified that measured the different dimensions of social vulnerability, met the indicator selection criteria, and had data available (Table 3). Given the importance of having local-level data for end users, the primary data source used was the 2013 New Zealand Census of Populations and Dwellings.

For measuring exposure, indicators were included about the population and ethnic groups, to provide context about the populations living in an area. Exposure indicators were also included relating to indirect impacts (such as people relying on public transport) and occupational exposure (such as people working in primary industries).

For some dimensions where no relevant data were available at the local level, proxy indicators were used, to ensure that each dimension had at least one indicator. Instead of using data on the number of pregnant women, the number of babies aged under 1 year was used as a proxy. Due to a lack of specific data for the food and water dimension, the following proxy indicators were used: households living in rental housing, single-parent households, and neighbourhood deprivation (New Zealand Index of Deprivation 2013) [63]. In New Zealand, these population groups are less likely to have household emergency preparedness [64], and are also more likely to experience household food insecurity (whereby households do not have sufficient food on a regular basis) [65]. For the decision-making dimension, no data were readily available at the very local level, so voting participation at the local council level was used instead. Health indicators relating to people with certain health conditions (such as cardiovascular disease, respiratory disease, and mental health issues) were not able to be completed within the timeframes of this study, but could be developed in the future.

For some dimensions, overlapping indicators were included (for example, 65+ years, 75+ years, 85+ years) in order to meet the varying data needs of end users. Additionally, indicators were included in all the dimensions that they were relevant for, which meant some indicators (such as NZDep) appeared in multiple dimensions.

### 4.3. Point Locations Relating to Social Vulnerability

In addition to indicators, relevant point locations for the different dimensions of social vulnerability were identified using the conceptual framework. These point locations included places that socially vulnerable people live or spend time at, and/or important places for these people. In particular, these locations included:

- Schools and early childhood centres;
- Rest homes;
- Health providers, including primary health care clinics, pharmacies and hospitals;
- Marae, which are meeting houses for local Māori iwi;
- Visitor accommodation and temporary housing.

Other sites of social and/or spiritual significance to Māori were also included, such as urupā (cemeteries). Appendix B contains a full list of point locations by social vulnerability dimension.

### 4.4. Design and Implementation of Indicators—Case Study of Porirua City

**Figure 4:**
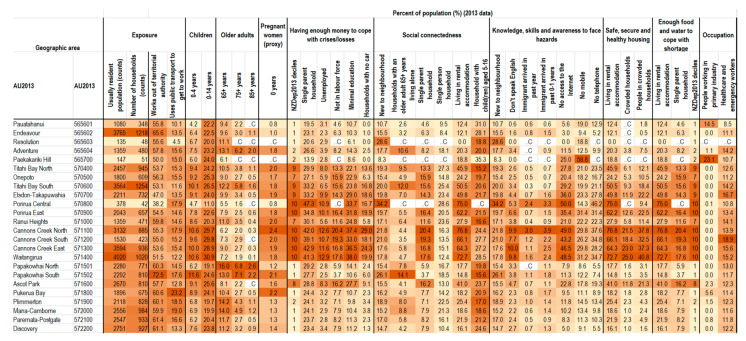
Social vulnerability indicators heatmap for Porirua City (2013 data by area unit (AU)) (more vulnerable areas are coloured darker) (screenshot). Note: .C denotes suppressed values due to low numbers. For the social connectedness indicator of households with child(ren) aged 5–16 years, higher values mean lower vulnerability, so lower values have been coloured darker.

The social vulnerability indicators were tested using the case study of Porirua City, with the heatmap of social vulnerability indicators highlighting some key geographic areas of vulnerability (Figure 4). Cannons Creek and Waitangirua had a higher level of vulnerability across multiple dimensions and indicators, including higher socioeconomic deprivation (scoring a 10 on the NZDep2013 decile, signifying the highest decile of deprivation), and higher levels of single-parent households, people not able to speak English, and households with no access to the internet. Other flood-prone areas, such as Elsdon-Takapūwāhia, Titahi Bay, Ascot Park and Porirua East also had higher levels of vulnerability across similar indicators, including single-parent households, socioeconomic deprivation, living in rental accommodation, and not having access to the internet. Porirua Central, while having a low population count, had high levels of people being new to the neighbourhood, and having no access to the internet, as well as higher levels of single-parent households and living in rental housing. In these neighbourhoods, local residents may not be aware of the risks, and, given their vulnerabilities, may have less capacity to prepare, cope and recover. Other areas, such as Papakowhai, were vulnerable primarily due to the susceptible populations (such as older adults) living there, and the higher percentage of people new to the area.

The heatmap also allowed understanding of specific vulnerabilities. For example, across most areas of Porirua, there is a high percentage of working adults who commute outside of Porirua City for work, and/or use public transport to get to work. This information could be useful for understanding impacts of floods, such as disruptions to transportation networks.

**Figure 5:**
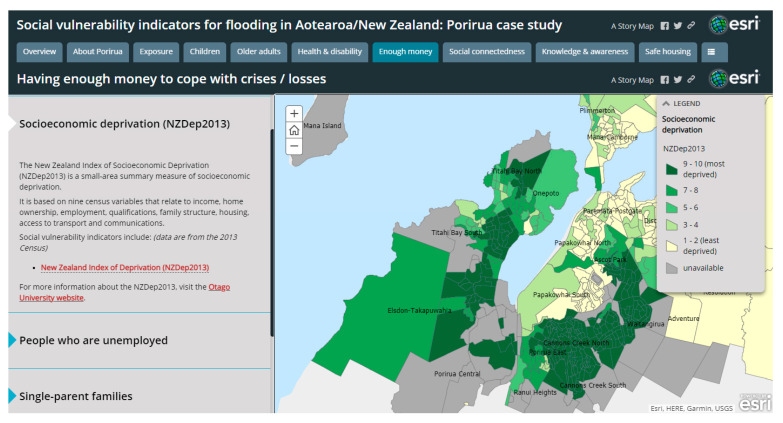
Screenshot of the online interactive map (Story Map) of social vulnerability indicators for Porirua, showing NZDep2013 at the meshblock level.

The social vulnerability indicators for Porirua City were also published on an online interactive map (Story Map), where individual indicators could be explored. For example, the online map of neighbourhood deprivation (NZDep2013 deciles) showed that many of the flood hazard zones were also areas with high deprivation, including Cannons Creek, Waitangirua, Porirua East and Takapūwāhia (Figure 5).

**Figure 6:**
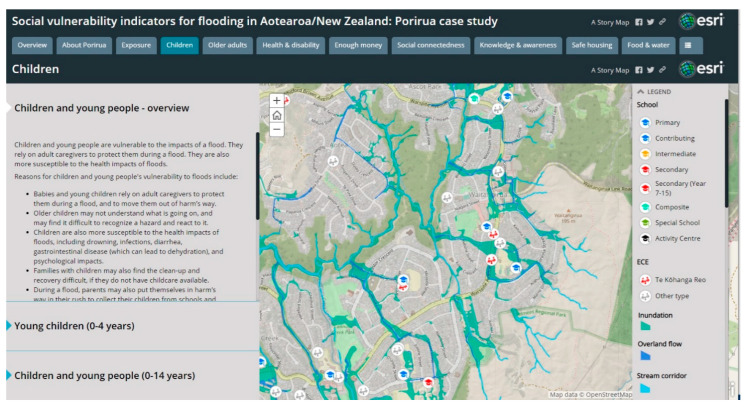
Screenshot of the online interactive map (Story Map) of flood hazard zones and school locations for Waitangirua, Porirua. Note: Flood hazard zones show 1-in-100 year flood extents, accounting for 100 year climate change impacts. Source: Wellington Water, Porirua City Council.

The social vulnerability indicators were complemented by maps of the flood hazard zones and locations of relevant point locations (such as schools and early childhood education centres (ECEs)). Figure 6 gives the example of Waitangirua, and shows that flood hazard zones affect much of the suburb, including several schools and early childhood education centres. The flood hazard and high levels of social vulnerability in this neighbourhood highlighted this geographic area as important for future risk reduction activities (such as flood management systems) and emergency preparedness and planning in order to meet the diverse needs of this neighbourhood.

### 4.5. Identifying Potential Uses for the Social Vulnerability Indicators

In response to these indicator outputs, stakeholders identified numerous ways that the social vulnerability indicators and data visualisations could be used. Firstly, the indicators could provide a structured way of thinking about and understanding social vulnerability to flooding, as well as objective measures of social vulnerability, before a flood event. Understanding the specific needs of vulnerable population groups could allow planning and readiness activities to be carried out in advance, to cater for these population groups. This could help support an equitable response to emergency preparedness, planning, response and recovery. The indicator data and data visualisations could also be used to spark discussion and initiate further data gathering at the local level.

During a flood event, social vulnerability indicators could provide information about the likely needs of the population for response activities, to help target and prioritise resources and efforts to areas with the highest needs, without needing to rely first on a ground survey. The indicators could also contribute to the development of shared situational awareness across the civil defence and emergency management response. After a flood, during the recovery phase, the indicators could help identify areas where people may need more support (e.g., financial support). Public health services and primary health care could also use the information to understand where socially vulnerable people live, to support health care provision.

The indicators could also support important up-stream risk reduction efforts, including infrastructure upgrades, hazard mitigation, and provision of resilient housing in areas with large numbers of socially vulnerable people. For example, having objective data about social vulnerability could allow decision makers to consider factors other than economic impacts when deciding stormwater infrastructure upgrades. An extension of this study also identified how land use planning could mitigate the impacts of natural hazards for vulnerable populations, through restricting development in areas subject to natural hazards, restricting the location of critical buildings and vulnerable land uses (based on the point locations identified in this study) in areas subject to natural hazards, and requiring urban design that promotes resilience (such as connectivity of routes for evacuation) [66].

## 5. Discussion

### 5.1. A New Set of Social Vulnerability Indicators for New Zealand

This study has implemented the first national set of social vulnerability indicators for flooding in New Zealand. These indicators provide objective measures of social vulnerability related to flooding to inform disaster risk management activities for New Zealand civil defence practitioners, local councils, and health professionals, and may also be of interest to local residents.

The indicators align with similar sets of social vulnerability indicators for flooding, with indicators about children, older adults, health status, poverty, lower levels of education, unemployment, lack of vehicle, single-parent households, rental housing, household crowding, and limited English proficiency, similar to previous sets [23,25,27,31,32,34,35]. The New Zealand indicator set also included different, relevant indicators about single-person households, recent immigrants, access to the internet, and people commuting outside of their local area for work, both to reflect the conceptual framework, and in response to feedback from stakeholders. The indicators also more generally reflect the vulnerable populations identified in the Sendai Framework for Disaster Risk Reduction 2015–2030 [67].

The indicators provide an important tool in managing disaster risk, by providing objective data about social vulnerability in the population to inform disaster risk reduction activities. For example, indicators about children, older adults, and people with health needs may be of particular interest to health providers, as these indicators represent populations who are more susceptible to health impacts. Indicators of poverty may show where welfare response and financial assistance is likely to be most helpful and could be prioritised during a response and recovery. Housing indicators may be useful when considering emergency housing during a response. Indicators about access to a motor vehicle, working outside of the area, and/or use of public transport, may be useful for understanding potential transportation and evacuation issues during a flooding event. Additionally, the point locations relating to social vulnerability can be combined with flood hazard zone information, to inform mitigation plans and support risk reduction activities, for example through land use planning, infrastructure upgrades and hazard mitigation works.

For the case study of Porirua, the indicators highlighted several critical geographic areas that had more vulnerable populations living in flood hazard zones, including Cannons Creek, Waitangirua, Elsdon-Takapūwāhia, Titahi Bay, Ascot Park, Porirua Central and Porirua East. Potential barriers to people developing resilience were identified, such as communities having a low level of internet access. This type of information could be used by civil defence emergency practitioners to guide how they interact with local neighbourhoods, for example by informing communications strategies.

More generally, indicator data are now also available for all areas of New Zealand, down to a small geographic scale, through online datasets and interactive online data visualisations. These datasets and data visualisations will allow end users to explore social vulnerability indicators for their local area, and allow similar assessments of vulnerability related to flood hazard to be carried out.

### 5.2. The Value of Using a Conceptual Framework to Guide Indicator Selection

Developing a conceptual framework for understanding social vulnerability was a crucial step in this study. While conceptual frameworks already existed for understanding vulnerability, resilience and capacities, this is the first time to our knowledge that these frameworks have been combined in this way to create a practical framework to guide selection of social vulnerability indicators for natural hazards. In particular, this study extends the application of the MOVE framework, to tease out what ‘lack of resilience’ may look like within the MOVE framework, in the context of social vulnerability. The use of well-established frameworks means that the conceptualisation of social vulnerability used in this study is robust and consistent with previous research. Using the MOVE framework also means the indicators are appropriate in the context of climate change.

Using a conceptual framework overcame several challenges often experienced when developing social vulnerability indicators. Firstly, the framework provides guidance on the topics that the indicators should cover, overcoming the difficulties of trying to directly replicate indicator sets from other parts of the world, which may not be relevant to New Zealand. Indicator topics in the framework include housing, social connectedness, knowledge of hazards, and economic wellbeing, as well as indicators about susceptible population groups, such as children, older adults, and people with health needs. While these topics are consistent with those included in other social vulnerability indicator sets, using the framework has the advantage of ensuring that the indicator set covers all dimensions of vulnerability.

Furthermore, the framework gives prominence to all aspects of vulnerability and resilience, regardless of data availability; each dimension is still represented as best it can with the data available. For example, decision making is highlighted as important in the circle of capacities [55] and the Sendai Framework, yet is often left out of social vulnerability indicator sets, or is indirectly represented through indicators of ethnicity, race or women. These variables are very country specific, and can reflect underlying vulnerabilities such as marginalisation, institutional racism, ongoing impacts of colonisation, and lack of representation in local body politics and emergency management groups. Recognising this with relevant indicators about ethnicity, race or women (in either the exposure or decision-making dimension), and/or wider variables such as voter turnout, can help reflect the underlying resilience factor of decision making.

The framework also provides a blueprint for ongoing development of indicators and improvements in data sources and data quality, and can highlight gaps in data sources. For example, when new data from the 2018 New Zealand Census of Populations and Dwellings became available after this study was completed, the framework could be used to guide indicator updates. When some indicators were unavailable to be updated due to a lack of data, replacement indicators could be selected to measure the same underlying dimensions of vulnerability. New indicators could also be identified to supplement existing indicators, for specific dimensions of social vulnerability.

### 5.3. Limitations and Challenges

One limitation of the indicator outputs from this study is that indicators are only *indicators* of reality, and therefore only show part of the picture of vulnerability and resilience for local communities. Additionally, in the response and recovery stages of a flood, the indicators will not give precise measurement of who is most affected, but they can provide initial information for action, before ground surveys can be carried out. The indicators also relate only to individual-level social vulnerability, rather than system-level factors, so will not include many factors that influence resilience at the system level. Nonetheless, these indicators can still be considered as some of the main factors contributing to individual-level social vulnerability, and can be supported by and interpreted in the context of local knowledge and supplementary data from local areas, as well as information about wider social, community-level, macro-level and structural factors that influence vulnerability.

Major challenges for these indicators included data availability and data quality. Some social vulnerability dimensions were difficult to obtain neighbourhood-level data for, and therefore the best available data were used (for example, using voter turnout data at the local council level for the decision-making dimension), which could be supplemented with other local data by end users. Furthermore, some potential indicators (such as health indicators about the prevalence of chronic diseases) were unable to be implemented within the study time period, but could be developed at a later date. Additionally, this study originally aimed to use 2018 Census data, but data delays meant that older 2013 Census data had to be used instead. Nonetheless, the indicator framework allowed for indicator updates once the 2018 Census dataset was made publicly available.

There were also some changes to personnel in our stakeholder group during the two-year flood study, due to work commitments and job changes. This meant that some people came into the study at a later stage, without the previous background. However, their input was still highly valuable, and provided different perspectives at different time points throughout the study.

More broadly, while having multiple indicators allowed end users to understand the underlying vulnerabilities in an area, this approach does not provide a succinct summary value for a geographic area. We have tried to overcome this issue by using heatmaps to visually show the indicators across the social vulnerability dimensions at a single glance. However, an index could be developed from these indicators in future, informed by end-user feedback on indicators that are the most useful across multiple hazards, and that have the greatest impact on interventions. This approach would ensure the index itself is grounded in experience and end-user decisions about dimensions of vulnerability.

### 5.4. Extending the Use of the Social Vulnerability Indicators

The approach used in this study could be applied in other jurisdictions to create social vulnerability indicators for flooding. The indicator development process provides a step-by-step guide for developing the indicators, with consideration of and input by stakeholders throughout the process. The conceptual framework could be used to guide indicator selection, with the dimensions of the framework prompting consideration of important topics, to ensure that these are all included as best as possible. While it is not expected that the New Zealand indicators would be directly useful in other countries, they could be used as a starting point, and adapted or replaced as needed.

This study has also laid the groundwork for a range of further work, in particular testing whether the framework and indicators could work for other hazards. Usefully, the conceptual framework has been developed for both natural hazards and climate change, and aligns with existing sets of social vulnerability indicators for natural hazards published internationally. A broad concept of vulnerability to natural hazards has been used, to incorporate the key elements of exposure, susceptibility, and lack of resilience, which are likely to apply to a range of hazards. Susceptible populations, such as children, older adults, and people with physical and/or mental health needs or a disability, will generally be more vulnerable to the negative impacts of any disaster. Furthermore, those people who lack resilience, in terms of a lack of money, social connectedness, hazard knowledge and skills, housing, emergency preparedness, and/or inclusion in decision making, are likely to be more vulnerable, regardless of the natural hazard. Given this, the framework and indicators may be relevant for other sudden-onset hazards, such as extreme weather events, wildfires, heatwaves, earthquakes and tsunami. Further work could test whether the framework would be appropriate across different hazards.

The indicators may also be useful for health emergencies, such as the infectious disease pandemic emergency of COVID-19. In the New Zealand context, potential impacts of COVID-19 include illness and death, as well as impacts from stress, lockdowns, and border closures (such as financial problems, job losses, potentially reduced access to health services and medication, mental health impacts, household crowding, and difficulties in accessing health services). Factors influencing high-risk groups in an influenza pandemic are similar to those for flooding, including poverty, single parents, substandard housing, crowded housing, children, immigrants, people with higher health needs or disabilities, older adults, and people with higher health needs, impaired immune systems or disabilities [68], as well as Māori and Pacific people in the New Zealand context [69]. Additionally, in countries with high levels of COVID-19, older adults aged 70 years and over appear to be at higher risk of experiencing severe illness and/or death if they contracted COVID-19. The similarity of vulnerability themes with the flooding indicators suggests that the social vulnerability indicators could be used for COVID-19 in New Zealand, with additional indicators about older adults aged 70 years and over, and people in occupations most likely to suffer economically due to the whole-of-society approach to managing the pandemic. Further work could test the utility of these indicators to the health sector.

Further work could also identify how well the indicators and framework work for longer-term climate-related disasters, such as drought and sea level rise. Some adaptations to the framework and additional indicators may be required, for example relating to people’s longer-term adaptive capacity to climate change. Future vulnerability could also be considered, for example exposure to projected future hazard zones (taking into account the impacts of climate change), projected population demographic trends, and projected trends in vulnerability. Future causes of vulnerability could also be considered, such as a lack of ability to get insurance in hazard zones in the future.

## 6. Conclusions

This study has successfully created and implemented a set of social vulnerability indicators for flooding for New Zealand. Differently from many previous sets of social vulnerability indicators, we have developed a conceptual framework for social vulnerability to guide indicator selection. We identified the following ten dimensions relating to social vulnerability: exposure; children; older adults; health and disability status; having enough money to cope with crises and losses; social connectedness; skills, knowledge and awareness of natural hazards; safe, secure and healthy housing; enough food and water to cope with shortage; and decision making.

This study has filled an important information gap in New Zealand, as no set of social vulnerability indicators for natural hazards or flooding had previously been fully developed and implemented in the New Zealand context. Together, the social vulnerability indicators and the point locations provide valuable information for action in New Zealand, for example to strengthen local planning for emergencies, and to inform risk reduction activities and response efforts during a disaster. The indicators allow a multi-disciplinary approach to disaster risk reduction, highlighting the importance of emergency preparedness, housing, and social connectedness to improving peoples’ resilience to natural hazards.

The approach used to develop these social vulnerability indicators could be applied in other jurisdictions, and could potentially be used with other hazards. In particular, the conceptual framework and indicators may work with other sudden-onset disasters, such as wildfires, heatwaves, earthquakes and tsunami, as well as pandemics such as COVID-19.

## Figures and Tables

**Table 1:** Selection criteria for selecting social vulnerability indicators.

Type	Selection Criteria	Details for the Social Vulnerability Indicators
Data sources	Data availability	Data need to be currently available; not too much work required to extract data
	Methodologically sound measurement	Data source needs to be reliable, accurate, and representative of the population; minimal bias and/or undercounting
	Able to be disaggregated	Data need to be available at a local level (neighbourhoods if possible)
	Timely	Data need to be collected and reported frequently, so that data are not too out of date
Measurement of indicators	Intelligible and easily interpretable	Indicators should be not too complex to understand, and should be able to be understood by a wide range of end users
	Methodologically sound measurement	Indicator definition and measurement needs to be robust, and should measure some aspect of the social vulnerability dimension
	Consistent with other indicator programmes	Indicators would ideally align with other indicator datasets already being used by end users
	Sensitive to change	Indicators are sensitive to change, so that they are measuring the current situation
	Comparable over time	Less of a priority for social vulnerability indicators, although ideally indicators can be interpreted in similar ways over time
Indicator relevance and appropriateness	Scientifically valid	Robust evidence needed for why the indicators are important (given the conceptual framework)
	Public health impact	Indicators need to relate to an issue of significant public health impact for the country; if numbers are too low nationally for an indicator, the indicator may not be very helpful.

**Table 2:** Dimensions of social vulnerability to natural hazards, and their rationale [16,17,23,24,25,61,62].

Social Vulnerability Dimension	Rationale
Exposure	Includes population groups exposed through direct impacts (such as living in a flood hazard zone), indirect impacts (such as through disruption to essential infrastructure and services, road transport networks, public transport, power supplies), and occupational exposure.
Children	Children rely on caregivers to protect them, and they may not understand the hazard or how to best react to it. Children are also more susceptible to certain health impacts, as they are still growing and developing.
Older adults	Older adults often have pre-existing health conditions, and may be less mobile and/or have vision or hearing loss. They may also experience social isolation.
People with health needs and/or disability	People with existing physical or mental health needs can be susceptible to the stress and physical impacts of natural hazard events, and may also be adversely impacted by disruptions to health services or medications. People with disabilities may need others to help them, and may have difficulties accessing emergency shelters.
Enough money to cope with crises and losses	People with low or no household income may not be able to afford preparedness measures (such as insurance, stockpiling food, medications and other resources), or be able to replace items after a natural hazard.
Social connectedness	Social isolation can leave people more vulnerable, as they may not have others to help them when needed. By contrast, strong social connections and support can provide resilience through networks and shared resources.
Knowledge, skills and awareness to face hazards	People are more vulnerable if they are not aware of hazards, not able to access information, or do not know how to prepare or react during a hazard event. Having knowledge, skills and awareness of hazards allows people to better prepare for and cope during a natural hazard.
Safe, secure and healthy housing	Shelter, warmth and security are basic human needs. People living in substandard housing, or in overcrowded housing, have increased risk of experiencing negative impacts of natural hazards, and may find it difficult to cope and recover.
Food and water to cope with shortage	Having sufficient food and water is a basic human need. After a natural hazard, having access to these resources (e.g., through household preparedness) is an important source of resilience. Not having enough emergency food (or enough food on a daily basis) or water (e.g., piped water) leaves people more vulnerable after a natural hazard.
Decision making and participation	Good leadership and decision making are vitally important before, during and after a natural hazard. Furthermore, people’s ability to access and participate in decision making, and the inclusion of a diverse range of people in decision making (especially vulnerable population groups and marginalised groups), is important for ensuring their needs are met in emergency management planning. Lack of involvement or inclusion in decision making can increase people’s vulnerability, as it means their needs may not be met or planned for through mitigation plans.

**Table 3:** Social vulnerability dimensions and indicators for flooding for New Zealand.

Dimension of Social Vulnerability	Social Vulnerability Indicators
Exposure (direct)	Number of people Number of households Ethnic groups (European, Māori, Pacific peoples, Asian, Middle Eastern/Latin American/African)
Exposure (indirect)	People who commute outside of the area People who use public transport to get to and from work People living in rural or remote communities
Exposure (occupational)	Health care workers and first responders People working in the primary industries
Children	Children aged 0–4 years Children aged 0–14 years
Older adults	People aged 65+ years People aged 75+ years People aged 85+ years
Health and disability	Pregnant women (proxy used of babies aged <1 year)
Having enough money to cope with crises/losses	Socioeconomic deprivation (NZDep2013 deciles) [63] Single-parent households Unemployed Not in labour force People with minimal education Households with no car
Social connectedness	People who are new to the neighbourhood (within previous year) Older adults (65+ years) living alone Single-parent households Single-person households Households living in rental housing Neighbourhoods with fewer households with children Recent immigrants
Knowledge, skills, and awareness of natural hazards	People who are new to the neighbourhood (within previous year) People with limited English proficiency Recent immigrants Households with no access to the internet Households with no access to a mobile phone Households with no access to a telephone
Safe, secure and healthy housing	Households living in rental housing Crowded households People living in crowded households People who are homeless and/or severely housing deprived *
Enough food and water (and other essentials) to survive	Households living in rental housing Single-parent households Socioeconomic deprivation (NZDep2013) [63]
Decision making and participation	Voter turnout in Local Authority elections *

* Data were only available at the territorial authority level. Note: Further indicator details are available in Appendix B.

## Data Availability

Social vulnerability indicator data for New Zealand for 2013 are available from the URL: https://www.ehinz.ac.nz/our-projects/social-vulnerability-indicators/ (Accessed on 9 April 2021). Indicator data for 2013 are available as downloadable Excel files for New Zealand, and online data visualisations for Porirua. Updated social vulnerability indicator data for New Zealand for 2018 are available from the URL: https://www.ehinz.ac.nz/indicators/population-vulnerability/social-vulnerability-to-natural-hazards/ (Accessed on 9 April 2021). Indicator data for 2018 are available as downloadable Excel files, and online data visualisations, for the whole of New Zealand.

## Appendix A

Comparison of dimensions of social vulnerability conceptual framework with previous sets of social vulnerability indicators.

**Table A1:** Previous sets of international social vulnerability indicators, by social vulnerability dimension.

Social Vulnerability Dimensions	Social Vulnerability Indicators for Natural Hazards			Social Vulnerability Indicators for Flooding		
	SoVI [27]	Social Determinants of Vulnerability Framework [25]	Social Vulnerability Index for Disaster Management [23]	MOVE Framework for Cologne Flood Vulnerability [33]	Urban Municipality Flood Vulnerability Index [31]	Social Flood Vulnerability Index (Flood Hazard Research Centre) [32]
Exposure	Occupation			Number of people living in flood-prone areas	Location of dwellings in low-lying coastal zone	
Children and older adults	Age	Children Older adults (65+ years)	0–17 years 65 years and over	Age structure (inability to evacuate)	Children (<15 years) Older adults (65+)	Residents aged 75+ years
Health and disability status		People with disabilities People with chronic and acute medical illness	People with a disability	Invalid (inability to evacuate)	Percent disabled Composite health indicator	Long-term sick—mobility problems (restriction in daily activities due to long-term illness, handicap or chronic disease)
Having enough money	Personal wealth	Low-to-no income Less than high school diploma Lack of vehicle	Living below poverty line Unemployment Per capita income No high school diploma Single parents No motor vehicle	Households with insurance against flood damages	Equivalised household income % below absolute poverty line % not working % with cars	Financially deprived people Unemployment among 16+ years Non-car ownership Non-house ownership No basis comfort Single parents
Social connectedness		Social isolation	Single parents			Single parents
Knowledge, skills and awareness of hazards		Limited English proficiency	Minority groups No high school diploma	Duration of residence (experience with floods)	% with mobile phone % with TV % with radio Distance of municipality to primary road	
Safe housing	Housing stock and tenancy	Renters	Multi-unit structures Mobile homes Household crowding		% owner occupier Presence of slums, tenements, informal settlements % of houses constructed with low-quality building material	Overcrowding Non-house ownership
Enough food and water					% of households with piped drinking water	
Other individual-level (exposure/decision making)	Race—African American, Asian Ethnicity—Hispanic, Native American	People of colour Women	Minority groups			
Outside of framework (structural/contextual)	Density of the built environment Single-sector economic dependence Infrastructure dependence		Living in group quarters	Performance of early warning system	Housing/land use plan indicating flood-prone areas Risk plan for environmental hazards Preparedness for floods % of households with sewage disposal system	

**Table A2:** Previous sets of social vulnerability indicators in New Zealand, by social vulnerability dimension.

Social Vulnerability Dimensions	Indicator Sets for Social Vulnerability		Indicator Sets for Resilience	
	SVIs for Earthquakes in NZ (main set) [35]	Vulnerability Assessment for the Hutt Valley [34]	New Zealand Resilience Index (NZRI) [48]	Bottom-up Approach for Neighbourhood-Based Resilience Framework [49]
Exposure		Population density Population growth Dwelling density	People not working in primary sector	
Children and older adults	Children living in married couple families Households receiving superannuation Population aged 0–4 years Population aged 65+ years Median age	Children aged 0–4 years Elderly aged 65+ years		
Health and disability status	Population living in nursing and skilled-nursing facilities Population with disability	Population on sickness benefit Population using NZ sign language	Hospitalisation rates	Pre-existing and post-disaster mental and emotional health of individuals
Having enough money	Poverty Households with no car Female-headed households Renters Unemployment Less than 12th grade education Employment in service industry Median house value Households earning greater than $XXX,XXX annually Per capita income Female participation in labour force	Single parents Having 3+ children No educational qualifications Household/individual income Receiving benefit Unemployed Working in sale and services, or primary industry No motor vehicle	People working in fulltime employment People with post-high school education Household income (equivalised)	Financial health of individuals, households and the neighbourhood
Social connectedness	Female-headed households Renters	Single parents Less than a year in current residence	Voluntary work Long-term residency	Collective efficacy Community participation Place attachment Social networks Social responsibility Stability of population Cultural values and practices
Knowledge, skills and awareness of hazards	Non-European Speaking English as a second language with limited English proficiency Less than 12th grade education	No telecommunications access Less than a year in current residence Living overseas 5 years ago English not first language	Long-term residency	Awareness of hazard risks Self-efficacy Community efficacy Education and training on responding to disasters Availability and accessibility of disaster risk information Diversity of skills Past experiences of disasters and other adverse events Understanding potential hazard impacts and consequences
Safe housing	People per unit	Population per dwelling Rental housing No fuel for heating	Number of emergency shelters per 1000 people	Structural integrity of buildings and infrastructure
Enough food and water			% of households with emergency water for three days	Personal responsibility for self-protection (e.g., disaster preparedness)
Other individual-level (exposure/decision making)	Female	Females Ethnic populations—Māori, Pacific, Asian, MELAA		
Outside of framework (structural/contextual)	Hospitals per capita		Economic sector diversity Infrastructure independency systemic resilience metric % commercial buildings that meet at least 34% of building standard Land use change between 1990 and 2012 Registered historic sites damaged/destroyed since 2000 % completeness of hazard planning from district plans Number of hospital beds per 1000 people Average distance to designated Community Emergency Response Centre	Neighbourhood space and amenities Civic infrastructure Experiences and effectiveness of collective action Unifying leadership Inclusiveness Community planning

## Appendix B. Social vulnerability indicators for New Zealand

**Table A3:** Social vulnerability indicators for flooding in New Zealand, full list, 2013 indicators.

Dimension of Social Vulnerability	Social Vulnerability Indicators	Data Source	Indicator Details
Exposure (direct)	Number of people	2013 Census	Usually resident population
	Number of households	2013 Census	Number of households
	Ethnic groups: (European, Māori, Pacific peoples, Asian, Middle Eastern/Latin American/African (MELAA)	2013 Census	Total response ethnic groups
Exposure (indirect)	People who regularly commute outside of the area	2013 Census	People with a work address located in different territorial authority (TA) to residential address
	People who use public transport to get to and from work	2013 Census	People who used public transport (train, bus, ferry) to get to work on Census day
	People living in rural or remote communities	2013 Census	People living in a rural centre or rural area
Exposure (occupational)	People working in the primary industries	2013 Census	People working in agriculture, forestry or fishing
	Health care workers and first responders	2013 Census	People working in health care and social assistance, police services, fire protection and other emergency services, and ambulance serivces
Children	Children aged 0–4 years Children aged 0–14 years	2013 Census	
Older adults	People aged 65+ years People aged 75+ years People aged 85+ years	2013 Census	
Physical health needs	People with a pre-existing health condition (including heart disease, diabetes, high cholesterol, respiratory conditions, immunosuppression)	To be developed	
	People requiring essential medications or health services (such as angina medication, insulin, inhalers, epilepsy medication, immunosuppressant drugs, anti-HIV drugs, dialysis, home oxygen therapy cancer treatment)	To be developed	
	Pregnant women	2013 Census	Proxy indicator used: Children aged <1 year
Mental health needs	People accessing mental health services in the past year	To be developed	
	People requiring essential medication for mental illness (anti-depressants, anti-anxiety medication, anti-psychotics, opioid substitution treatment)	To be developed	
	People with substance abuse issues	To be developed	
Disability	People with a disability (mobility, hearing, vision, learning/language, limited intellectual skills)	To be developed	
Having enough money to cope with crises/losses	Socioeconomic deprivation	Atkinson et al. [63]	New Zealand Index of Deprivation 2013 (NZDep2013) deciles
	Single-parent households	2013 Census	Households with one parent with child(ren)
	Unemployed	2013 Census	People who were unemployed, among those aged 15+ years
	Not in labour force	2013 Census	People who were not in the labour force, among those aged 15+ years
	People with minimal education	2013 Census	People with no qualification, among those aged 15+ years
	Households with no access to car	2013 Census	Households with no motor vehicle
Social connectedness	People who are new to the neighbourhood (e.g., within previous year)	2013 Census	Years at usual residence <1 year
	Older adults living alone	2013 Census	Adults aged 65+ years living in a single-person household
	Single-parent households	2013 Census	Households with a single parent and dependent children
	Single-person households	2013 Census	Households with only one person
	Households living in rental housing	2013 Census	Dwellings not owned and not held in family trust
	Neighbourhoods with fewer households with children	2013 Census	Households with one or more children*
	Recent immigrants	2013 Census	People who moved to New Zealand recently (<1 year, <2 years)
Knowledge, skills, and awareness of natural hazards	People who are new to the neighbourhood (within previous year)	2013 Census	Years at usual residence <1 year
	Households with no access to a mobile phone	2013 Census	Households that did not have access to a mobile phone
	Households with no access to the internet	2013 Census	Households that did not have access to the internet
	People with limited English proficiency	2013 Census	People who reported not speaking English
	Recent immigrants	2013 Census	People who moved to New Zealand recently (<1 year, <2 years)
Safe, secure and healthy housing	Households living in rental housing	2013 Census	Dwellings not owned and not held in family trust
	Crowded households	2013 Census	Households that needed 1+ bedrooms, according to the Canadian National Occupancy Standard
	People living in crowded households	2013 Census	People living in households that needed 1+ bedrooms, according to the Canadian National Occupancy Standard
	People in severe housing deprivation (homelessness)	Amore et al. 2016 [70]	People who were severely housing deprived (or homeless) in 2013 *(only available at territorial authority level)*
Enough food and water (and other essentials) to survive	Households living in rental housing	2013 Census	Dwellings not owned and not held in family trust
	Single-parent households	2013 Census	Households with a single parent and dependent children
	Socioeconomic deprivation	Atkinson et al. [63]	New Zealand Index of Deprivation 2013 (NZDep2013) deciles
Decision making and participation	Voter turnout in local elections	NZ Department of Internal Affairs	Residential voter turnout in the 2016 Local Body elections * *(only available at territorial authority level)*

* Indicator relates to positive impact, so higher values should be interpreted as lower vulnerability. For all other indicators, higher values relate to higher vulnerability.

**Table A4:** Point locations relating to social vulnerability for flooding for New Zealand.

Dimension of Social Vulnerability	Point Locations Examples
Exposure (direct)	Emergency shelters
Exposure (indirect)	Main/arterial roads Public transport networks (bus routes, train tracks, train stations) Fire stations, police stations, ambulance stations Important utilities (power substations, water pumping stations, etc.) Hazardous substances facilities and contaminated sites
Children	Schools Early childhood education centres
Older adults	Rest homes Social housing for older adults
Physical health needs	Primary health care facilities Pharmacies Hospitals Medical supply depots Other health facilities (dialysis units, birthing units, long-stay hospitals)
Mental health needs	Mental health services Primary health care facilities Pharmacies Hospitals
Disability	Community residential homes Respite care facilities Specialist schools for children with disabilities and high needs
Having enough money to cope with crises/losses	Social housing Hazard areas where properties are not able (or prohibitively expensive) to be insured
Social connectedness	Schools Early childhood centres Churches Local meeting places (such as marae)
Knowledge, skills, and awareness of natural hazards	Visitor accommodation, such as motels, hotels, camping grounds Refugee settlement centres and locations
Safe, secure and healthy housing	Houses in flood hazard zones Emergency housing (night shelters, women’s refuge) Temporary accommodation (camping grounds, boarding houses, etc.)
Enough food and water (and other essentials) to survive	Food stores Food banks Local emergency water supplies
Decision making and participation	Marae Community Emergency Hubs
Group quarters and/or institutions (related to housing)	Prisons and youth justice facilities Community correction centres University dorms Military quarters
